# Intelligent Deep Models Based on Scalograms of Electrocardiogram Signals for Biometrics

**DOI:** 10.3390/s19040935

**Published:** 2019-02-22

**Authors:** Yeong-Hyeon Byeon, Sung-Bum Pan, Keun-Chang Kwak

**Affiliations:** Department of Control and Instrumentation Engineering, Chosun University, Gwangju 61452, Korea; qasdfghjt@hanmail.net (Y.-H.B.); sbpan@chosun.ac.kr (S.-B.P.)

**Keywords:** biometric, deep learning, electrocardiogram, comparative analysis, scalogram

## Abstract

This paper conducts a comparative analysis of deep models in biometrics using scalogram of electrocardiogram (ECG). A scalogram is the absolute value of the continuous wavelet transform coefficients of a signal. Since biometrics using ECG signals are sensitive to noise, studies have been conducted by transforming signals into a frequency domain that is efficient for analyzing noisy signals. By transforming the signal from the time domain to the frequency domain using the wavelet, the 1-D signal becomes a 2-D matrix, and it could be analyzed at multiresolution. However, this process makes signal analysis morphologically complex. This means that existing simple classifiers could perform poorly. We investigate the possibility of using the scalogram of ECG as input to deep convolutional neural networks of deep learning, which exhibit optimal performance for the classification of morphological imagery. When training data is small or hardware is insufficient for training, transfer learning can be used with pretrained deep models to reduce learning time, and classify it well enough. In this paper, AlexNet, GoogLeNet, and ResNet are considered as deep models of convolutional neural network. The experiments are performed on two databases for performance evaluation. Physikalisch-Technische Bundesanstalt (PTB)-ECG is a well-known database, while Chosun University (CU)-ECG is directly built for this study using the developed ECG sensor. The ResNet was 0.73%—0.27% higher than AlexNet or GoogLeNet on PTB-ECG—and the ResNet was 0.94%—0.12% higher than AlexNet or GoogLeNet on CU-ECG.

## 1. Introduction

Biometrics is a technology that automatically determines the identity of people using their physical and behavioral characteristics. Smartphones and IoT (Internet of Things) devices have become widespread, and the performance of their firmware has improved, so that they have been able to cover high computational load by themselves. Moreover, by receiving only results against the request for complicated analysis from a server through the Internet, various services can be implemented. If a service provider responds to requests indiscriminately, it may be exploited for crime, so it should be limited to the authorized person, and the required technology is authentication. Authentication methods of security-dependent services, such as access to private clouds and payments using smartphone banking, are moving toward applying convenient biometrics [1,2,3,4,5,6].

The characteristics used in biometric study include face [7], iris [8], gait [9], sweat pore [10], finger-vein [11], palmprint, palmvein [12], voice [13], handwritten signature [14], and electrocardiogram (ECG) [15]. The disadvantages of each characteristic include the face having restrictions on glasses, mask, and makeup, and the original face for recognition should be recovered. It is likely to be exploited for crime because it is vulnerable to camouflage. The iris has to be put close to a camera to be captured, and some glasses on faces are constrained for recognition. In order to measure the iris well, the eyes should be wide open, and the shape of iris varies according to the focus of the eyes. Using sweat pore, fingerprint, or palmvein as characteristics can be uncomfortable depending on the situation, because the hand cannot be used while measuring the data. In the case of gait, a person must perform a series of actions according to the scenario to obtain data. The voice is difficult to use in a noisy environment, and there is the inconvenience that a person must intentionally vocalize. The handwritten signature needs a pen to enter data, the hand is busy during writing, and the hand-written signature can be easily intentionally copied. The electrocardiogram is sensitive to the state and noise of the measuring signal [7,8,9,10,11,12,13,14,15].

The electrocardiogram is a measure of the change in voltage during the cardiac cycle. This change has uniqueness, because each person has a physically different body shape [16]. Recently, biometrics using the electrocardiogram has been carried out, and has shown good performance [17,18,19]. The complexity of the signal measurement process has been simplified, and measuring devices have been evolving into small, lightweight products in wearable form. Because the electrocardiogram signal is linked to the health check function, it is highly utilized. It is also advantageous for security, because the heart-generated signal is concealed inside the body. Through the sensor of the individual device, it is possible to measure without constraint anytime and anywhere. Biometrics using the electrocardiogram is sensitive to the state of the body and noise at measure, but studies are being conducted to solve these problems [16].

Since biometrics using electrocardiogram signals are sensitive to noise, studies have been conducted by converting signals into the frequency domain. Commonly used methods for the frequency analysis of signals are discrete cosine transform (DCT), continuous wavelet transform (CWT), and discrete wavelet transform (DWT). Li and Zhou performed electrocardiogram classification using wavelet packet entropy and random forests [20]. DCT is a linear combination of cosine functions with different frequencies that represent a signal and can compress the signal. Khorrami and Moavenian classified the electrocardiogram using DCT coefficients as a feature. This was compared with the classification results of features using CWT and DWT [21]. Song et al. used wavelet transform to extract 17 features from electrocardiogram signals, and features were used after dimensional reduction through linear discriminant analysis (LDA) [22]. Yu and Chen used some statistical features after decomposing the signal into sub-bands, using two-level DWT [23]. Ye et al. applied principal component analysis (PCA) to RR and features extracted from electrocardiogram by DWT and ICA (independent component analysis) [20,24]. Normally, the results of those transforms are 2-dimensional with axes of time and frequency. As studies to try signal processing in two dimensions, Soualhi et al. changed the electrocardiogram into a synchronous representation, then rearranged it every period to construct a two-dimensional image [25]. Zhai and Tin constructed a dual beat coupling matrix with beat morphology and beat-to-beat correlation, using two adjacent electrocardiogram beats [26].

In recent years, deep learning has been developed, breaking many existing limitations, and still, further improved methods are being proposed. Deep learning [27] is a neural network in which various types of layers are added, and many layers are piled up starting from the existing multilayer perceptron [28] in machine learning, and the problems that could not be solved with a small number of layers are solved by deep layers. There are deep neural networks [29], convolutional neural networks (CNN) [30], recurrent neural networks [31], deep belief networks [32], deep Q-networks [33], and so forth. One of the most prominent methods in image processing is a convolutional neural network that is designed to automatically learn various filters for feature extraction, and automatically classify the feature vectors. In the past, feature extraction was originally designed by humans, and machine learning was used only for classification. However, the convolutional neural network automatically learns not only classifiers, but also good features [34]. There are deep models with good performance in the ILSVRC (ImageNet Large Scale Visual Recognition Challenge) using the deep convolutional neural network [35,36,37]. When training data is small or hardware is insufficient for training, transfer learning can be used with pretrained deep models to reduce learning time and classify it well enough [38]. AlexNet [35], GoogLeNet [36], and ResNet [37] are existing deep models. AlexNet, which is similar to the existing LeNet [39], consists of seven layers and it is paralleled using two graphics processing units (GPUs) [35]. GoogLeNet is made up of 22 layers, and nine inception modules are applied [36]. ResNet is made up of 152 layers, and is added with residual learning, which reduces the time that is required to learn deep network, and enables successful learning, although it is very deep [37].

By transforming signal from time domain to frequency domain using the wavelet, the 1-D signal becomes a 2-D matrix, and it can be analyzed on multiresolution. However, this process makes morphologically complex signal analysis. This means that existing simple classifiers could be poor in performance. We investigated the possibility of using the image of frequency as input to CNN of deep learning, which exhibits optimal performance for the classification of morphological imagery. The work that compares the performance of deep models with input of the 2-D matrix using wavelet transform from ECG signals has not been studied so far. Thus, we focus on develop the most representative popular models frequently used in conjunction with deep learning models to perform ECG-based biometrics. The frequency image is here termed scalogram. AlexNet, GoogLeNet, and ResNet are considered deep models of the convolutional neural network. The novelty lies in the usage of scalogram to represent ECG features and testing its strength in biometric classification. Testing this method of input features in the form of image is appreciated for biometrics as time series of ECG signal does not visibly represent any differences regarding biometric analysis. The experiments were performed on two databases for performance evaluation. Physikalisch-Technische Bundesanstalt (PTB)-ECG is a well-known database, while Chosun university (CU)-ECG is directly built for this study using the developed ECG sensor. The ResNet was 0.73%—0.27% higher than AlexNet or GoogLeNet on PTB-ECG—and the ResNet was 0.94%— 0.12% higher than AlexNet or GoogLeNet on CU-ECG.

In this paper, we performed comparative analysis of deep models in biometrics using scalogram of electrocardiogram. Section 2 describes deep models of convolutional neural network, while Section 3 discusses the biometrics method using scalogram of electrocardiogram. Section 4 presents the experimental results, while Section 5 concludes the paper.

## 2. Deep Models of Convolutional Neural Network

### 2.1. AlexNet

**Figure 1:**
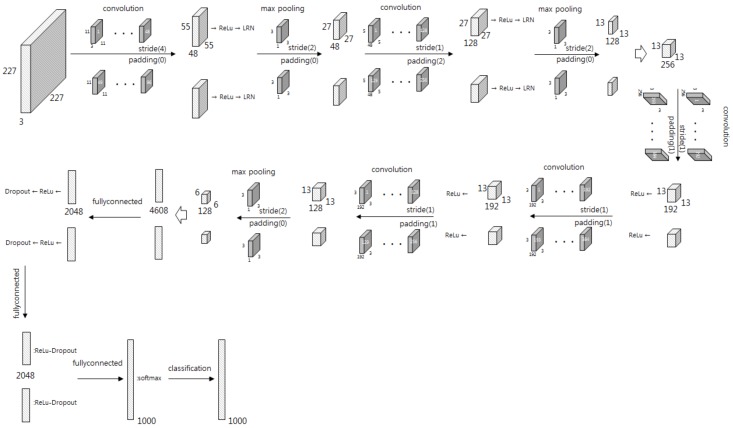
Structure of AlexNet.

AlexNet is a structure of deep learning applied in parallel to two GPUs, with five convolutional layers, three pooling layers, two fully connected layers, and an output layer. The input image must be converted to a size of 227 × 227. Figure 1 shows the structure of AlexNet. The input image is passed in parallel to calculate the convolution with 96 filters of 11 × 11 × 3 and a 4-stride, so two feature cubes of 55 × 55 × 48 are created. Next, ReLU (Rectified Linear Unit) of an active function and LRN (Local Response Normalization) are applied to the cubes. Max-pooling with filter of 3 × 3 × 1 and 2-stride for each cube reduces the dimension to 27 × 27 × 48 each. The cubes become 27 × 27 × 128 each using convolution with 256 filters of 5 × 5 × 48, 1-stride, and 2-padding, and are applied by ReLU and LRN. The cubes become 13 × 13 × 128 each by max-pooling with filter of 3 × 3 × 1 and 2-stride. Their concatenated cube of 13 × 13 × 256 becomes two 13 × 13 × 192 cubes in parallel by convolution with 192 filters of 3 × 3 × 256, 1-stride, and 1-padding twice, and then ReLU is applied. The cubes become 13 × 13 × 192 each by convolution with 192 filters of 3 × 3 × 192, 1-stride, and 1-padding, and then ReLU is applied. The cubes become 13 × 13 × 128 by convolution with 128 filters of 3 × 3 × 192, 1-stride, and 1-padding. The cubes become 6 × 6 × 128 by max-pooling with filter of 3 × 3 × 1, 2-stride, then are reshaped to 4608 × 1 as a one-dimensional vector. This is mapped to 2048 nodes, and then ReLU and dropout are applied, and that is repeated once more. Then it maps to 1000 nodes, which means the number of classes, and then softmax is applied for classification [35].

### 2.2. GoogLeNet

**Figure 2:**
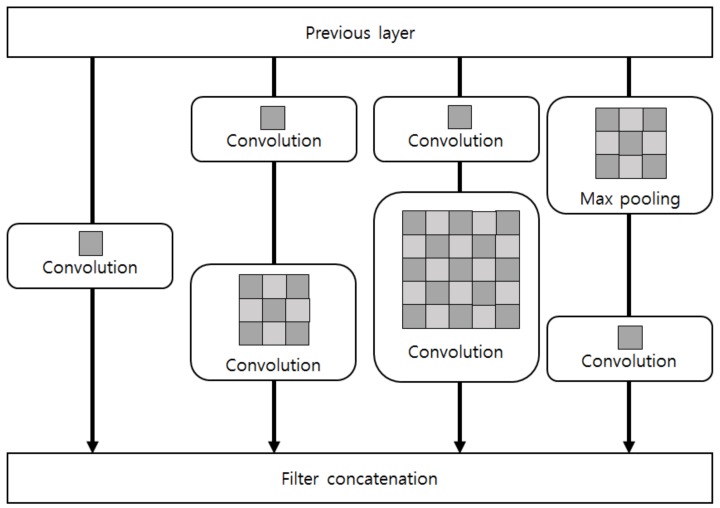
Structure of inception module.

GoogLeNet consists of nine inception layers. The idea of an inception layer is to keep the resolution good for small information, while covering a larger area in the image. Therefore, convolutions are performed at various sizes in parallel, from 1 × 1, which is the most accurate, to 5 × 5. Figure 2 shows the structure of the inception module. In inception, the convolution with filter of 1 × 1 reduces the number of feature maps, thereby reducing the amount of computation. Thus, the inception performs 1 × 1 convolutions first, and then performs convolutions of different sizes. In addition, max-pooling has been added to summarize the contents of the previous layer, and all the results are concatenated on the next layer. Also, two auxiliary classifiers were used in the middle of GoogLeNet structure, to avoid the vanishing gradient problem during learning [36].

### 2.3. ResNet

**Figure 3:**
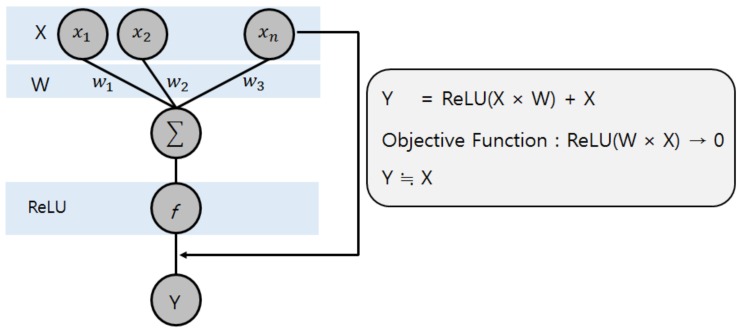
Principle of residual learning.

As the layer of the neural network becomes deeper, the problems of vanishing gradient, exploding gradient, and degradation occur. The vanishing gradient means that the gradient of the propagation becomes too small, while the exploding gradient means that the gradient of the propagation becomes too big, which makes it fail at training. Degradation means that deep neural networks perform worse than shallow neural networks, although no overfitting has occurred. ResNet improves the learning efficiency by reusing the input features of the upper layer to solve this problem. Figure 3 shows the principle of residual learning. The input of X makes the output of Y, and then the input of X is added to the output of Y again. Then, learning is performed for ReLU (W × X) to converge on 0, and this means that the output of Y is almost the same value as X. This is not a problem even if the intermediate weights are small, and small changes in the input are also reflected in the output. The number of intermediate weight layers can be placed randomly, and ResNet is a model that is stacked deeply in this way. Its basic framework is referred to VGGNet [40], it uses convolution only with a filter of 3 × 3 and does not use pooling or dropout. To reduce the size of the feature map, it uses convolution with 2-stride instead of pooling. Every two convolutions, the upper input layer is reused in the output [37].

## 3. Biometric Method using Scalogram of Electrocardiogram

The biometric method is performed with 3 steps in this paper. First, the 1-D signals of electrocardiogram are preprocessed to eliminate noises. Secondly, the preprocessed signals become 2-D scalograms using CWT and Morse wavelet. By transforming the ECG signal from the time domain to the frequency domain using the CWT and Morse wavelet, it can be analyzed on multiresolution. Thirdly, deep models are trained with input of the scalograms.

### 3.1. Preprocessing

Noise occurs when measuring the ECG signal, which is caused by low-frequency noise due to muscle movement and high-frequency noise of 50 to 60 Hz, depending on the power source used [41]. Low-frequency noise is removed by a convolution using an average filter of 500 filter size, and high-frequency noise is removed by convolution using an average filter of 10 filter size. Six-hundred frames are removed at the beginning and end of the signal, to remove the distorted parts due to filtering. After performing R-peak detection, 784 frames are obtained around the R-peak point. Only the I-lead of electrocardiogram was considered in this study [42].

### 3.2. Continuous Wavelet Transform

The wavelet transform is a more powerful method than the conventional cosine and Fourier transforms as a time–frequency transform [20]. Morlet introduced a wavelet as a family of translations and dilatations from a single function called the mother wavelet. This new signal processing has been improved more efficiently by Mallat, Meyer, Daubechies, and Grossman, and has become a popular technique in biosignal analysis. Instead of analyzing the Fourier transforms in a single scale (time or frequency), wavelet transforms are analyzed on a multiscale basis.

Wavelet is a windowing method with different resolutions for regions. Wavelet decomposition maps a signal into a time-scale plane using a scale instead of a frequency. This is the same as the time–frequency plane in the short-time Fourier transform (STFT) and each scale of the time-scale plane represents a certain frequency range of the time–frequency plane. A wavelet—a small waveform—is a localized wave for a limited duration. Comparing the wavelet with the Fourier transform, the Fourier analysis decomposes the signal into sinusoids of different frequencies, while the wavelet decomposes the signal into the shifted or scaled shapes from a mother wavelet.

The continuous wavelet transform (CWT) for the signal f(t) is defined as integration of the f(t) with the shifted or scaled shapes from a mother wavelet $\psi_{a,b}\left(t\right)$:

(1) $$\mathrm{CWT}\left(a,b\right)=\frac{1}{\sqrt{a}}\overset{+\infty }{\underset{-\infty }{\int }}f\left(t\right)*\psi (\frac{t-b}{a})dt\;a\in R^{+}-\left\{0\right\},\;b\in R$$

In other words, the CWT is the sum of the signal multiplied by shifted and scaled shapes from a mother wavelet ψ:

(2) $$\mathrm{CWT}\left(scale,position\right)=\overset{+\infty }{\underset{-\infty }{\int }}f\left(t\right)*\psi \left(scale,position,t\right)dt$$

The original basic wavelet ψ(t) is called the mother wavelet, and its variations $\psi_{a,b}\left(t\right)$ are called daughter wavelets. The daughter wavelets are the shifted or scaled shapes from a mother wavelet. The ‘*a*’ is scale factor for scaling the function ψ(t), while the ‘*b*’ is a shift factor for translating the function ψ(t). The result of the CWT is a matrix filled with wavelet coefficients located by scale and position. To determinate scale parameter and mother wavelet in CWT is very important for analyzing ECG [21].

### 3.3. Morse Wavelet

A wavelet means a small waveform satisfying two conditions. The first is that the amplitude of the signal rapidly decreases and converges to zero, and the second is that the function should oscillate. The wavelet filter has a feature that can decompose a signal into several frequencies and remove a specific frequency and amplitude. At this time, the role of the mother wavelet is important. There are several functions as mother wavelet. When the most similar wavelet with the signal to be decomposed is used, better noise cancellation without distortion can be performed [43].

A complex-valued wavelet in which the Fourier transform is only valid on the positive real axis is called an analytic wavelet, and a generalized Morse wavelet is its family. This is useful for analyzing signals with varying amplitude and frequency over time and localized discontinuities [44]. There have been many studies on Morse wavelet theory and its application to signal analysis [45,46,47], resulting in an efficient algorithm for calculating the Morse wavelet [48]. The Fourier transform of the generalized Morse wavelet is

(3) $$\Psi_{P,\gamma }\left(\omega \right)=U\left(\omega \right)a_{P,\gamma }\omega^{\frac{P^{2}}{\gamma }}e^{-\omega^{\gamma }}$$

where, U(ω) is the function of unit step, $P^{2}$ is the time–bandwidth product, $a_{P,\gamma }$ is a constant for normalization, and γ is a parameter for determining the symmetry of the Morse wavelet. In many applications of the Morse wavelet, β is used as a decay or compactness parameter, rather than the time–bandwidth product, $P^{2}=\beta \gamma$ [45]. The equation for the Morse wavelet using β and γ as parameters is

(4) $$\Psi_{\beta ,\gamma }\left(\omega \right)=U\left(\omega \right)a_{\beta ,\gamma }\omega^{\beta }e^{-\omega^{\gamma }}$$

Various analytic wavelets could be obtained by varying the time–bandwidth product and symmetry parameters of a Morse wavelet. Many useful analytic wavelets are a case of generalized Morse wavelets, such as Cauchy wavelets with γ=1 and Bessel wavelets with β=8 and γ=0.25.

**Figure 4:**
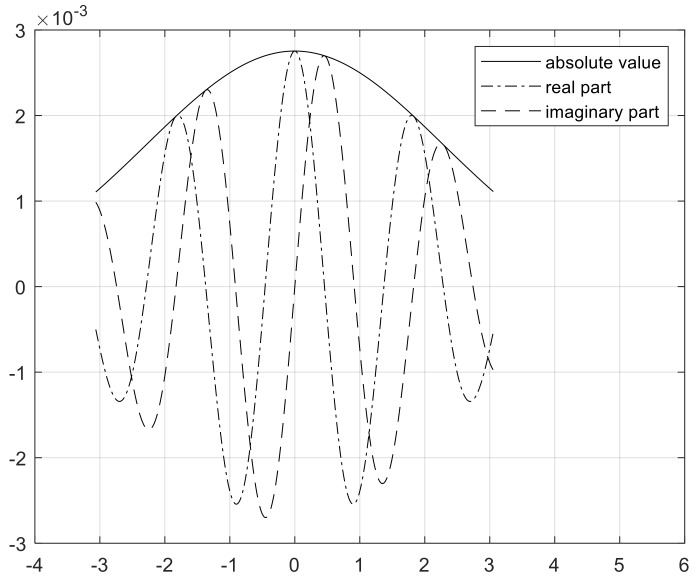
Morse wavelet with γ=3 and $P^{2}=60$.

The wavelet duration in time is proportional to the square root of *P*, which is the time–bandwidth product. The duration affects the number of oscillations of the center window at its peak frequency, ${\left(\frac{P^{2}}{\gamma }\right)}^{\frac{1}{\gamma }}$. The skewness of the Morse wavelet by demodulation is 0 when γ is 3 as the minimum Heisenberg area. Therefore, the value is used in this study [49]. Figure 4 shows the Morse wavelet with γ=3 and $P^{2}=60$. Its signal length, sampling frequency, and voices per octave are 784, 128, and 12, respectively. The number of voices per octave is used for discretizing the scales of CWT.

### 3.4. Biometrics using Scalogram of Electrocardiogram

**Figure 5:**
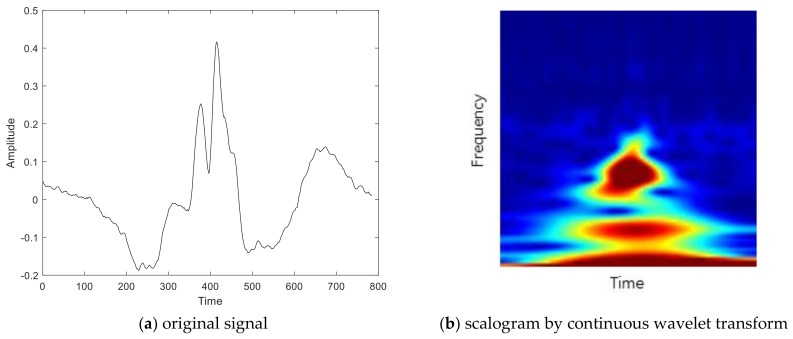
Scalogram of an electrocardiogram.

**Figure 6:**
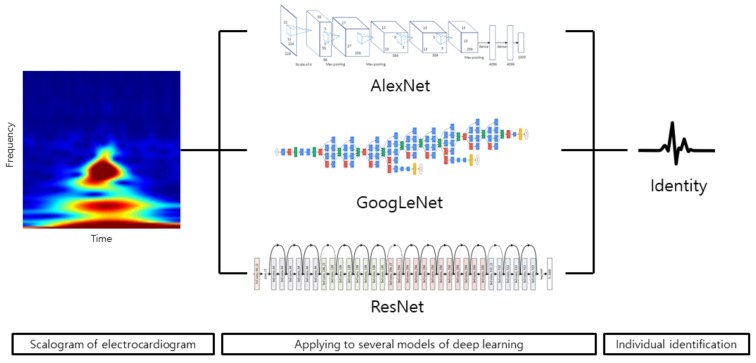
Biometrics using the scalogram of an electrocardiogram under varying the models of deep learning.

Since biometrics using electrocardiogram signals are sensitive to noise, studies have been conducted by converting signals into the frequency domain. Commonly used methods for the frequency analysis of signals are DCT, CWT, and DWT. A scalogram is the absolute value of the CWT coefficients of a signal. By transforming the signal from the time domain to the frequency domain using the wavelet the 1-D signal becomes a 2-D matrix, and it could be analyzed on multiresolution. The representation of the signal in the time domain is limited because it cannot represent the signal until infinite time. At this time, frequency–time analysis allows us to know how the signal is distributed with frequency and phase, so complex signals can be expressed concisely and analyzed easily. In other words, since the time-domain signals are noise and complex, different ECG signals may not be distinguishable due to lack of discriminatory features. However, by visually representing signals at various scales and various frequencies through CWT, hidden features can be seen in the frequency–time domain. The Morse wavelet with r=3 and $P^{2}=60$ is used as mother wavelet. However, this process makes morphologically complex signal analysis. This means that existing simple classifiers could be poor in performance. We investigate the possibility of using the image of frequency as input to CNN of deep learning, which exhibits optimal performance for the classification of morphological imagery. Figure 5 shows the scalogram of an electrocardiogram. Figure 6 shows biometrics using the scalogram of an electrocardiogram under varying the models of deep learning.

Here, accuracy is used to check the evaluation of the performance for biometrics. It is calculated by dividing the number of correct classification (CC) by the total number of classifications, the sum of CC, and the wrong classification (WC). The accuracy is defined as follows [50]

(5) $$\mathrm{Accuracy}=\frac{\mathrm{CC}}{\mathrm{CC}+\mathrm{WC}}$$

In the verification stage, accuracy, sensitivity, specificity, false positive rate (FPR), and false negative rate (FNR) are considered as evaluation methods. True positive (TP) and true negative (TN) mean correctly classified as positive or negative. False positive (FP) and false negative (FN) mean incorrectly classified as positive or negative. Those methods are defined as follows [50]

(6) $$\mathrm{Accuracy}=\frac{\mathrm{TP}+\mathrm{TN}}{\mathrm{TP}+\mathrm{TN}+\mathrm{FP}+\mathrm{FN}}$$

(7) $$\mathrm{Sensitivity}=\frac{\mathrm{TP}}{\mathrm{TP}+\mathrm{FN}}$$

(8) $$\mathrm{Specificity}=\frac{\mathrm{TN}}{\mathrm{TN}+\mathrm{FP}}$$

(9) $$\mathrm{FPR}=\frac{\mathrm{FP}}{\mathrm{TN}+\mathrm{FP}}$$

(10) $$\mathrm{FNR}=\frac{\mathrm{FN}}{\mathrm{TP}+\mathrm{FN}}$$

## 4. Experimental Result

### 4.1. Database

**Figure 7:**
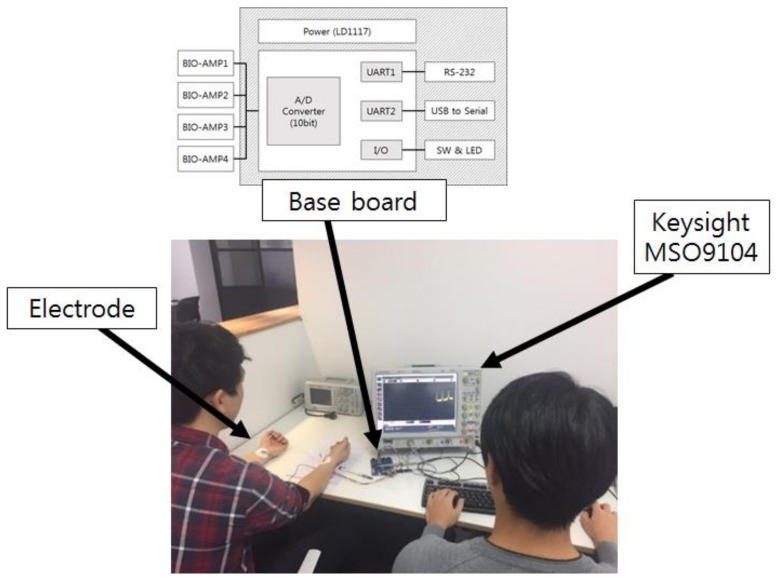
Developed device and environment for measuring ECG signals.

Two databases were used for comparative analysis of deep models in biometrics using the scalogram of electrocardiogram. First, PTB-ECG is ECG data obtained from the National Metrology Institute of Physikalisch-Technische Bundesanstalt (PTB) in Germany, and contains 27,000 recordings. This PTB-ECG was obtained from 290 people sitting comfortably, including males and females. Some people had heart disease, and signals were measured from 15 leads at 1000 samples/s. The 15 leads are composed of 12 standard leads and three Frank leads. Each person has a different number of recordings, usually recorded two or three times, varying in length from 23 seconds to 2 minutes. After measuring the ECG once per person, the next measure was performed after 500 days on average [51,52]. Secondly, the CU-ECG is ECG data directly built for the biometrics at Chosun university (CU) in Korea. This database is obtained from 100 subjects (11 females and 89 males ranging from 23 to 34 years old). Sixty recordings per person were measured while subjects were sitting comfortably in a chair. The data were recorded for 10 s at a time, and only the lead-I signal was measured. The sampling rate of measure was 500 kHz. The measuring was performed using a directly developed device. The developed device consists of Keysight MSO9104 (Keysight technologies, Santa Rosa, CA, USA), Atmega8 (Microchip technology, Chandler, AZ, USA), and a wet-corrosion electrode. Figure 7 shows the developed device and environment for measuring ECG signals [15].

### 4.3. Experimental Results

The computer specification used in the experiment is Intel(R) Xeon(R) E5-1650 CPU (Central Processing Unit) v3 at 3.50 GHz, NVIDIA GeForce GTX Titan X, 32 GB RAM (Random Access Memory), and Window 7 64-bit OS (Operating System). In this study, the biometric of the electrocardiogram was performed by generating a scalogram from raw signal. Signals were preprocessed to eliminate noise, and then R-peak points were detected for all recordings. The number of R-peak points were identified for each class. To construct the same count of data, classes consisting of small R-peak points were excluded, and classes consisting of many R-peak points abandoned some detected R-peak points. Seven-hundred-and-forty-eight frames were obtained around the R-peak point, only near the I-lead of the electrocardiogram. The preprocessed ECG signals were then transformed to the scalograms using CWT and Morse wavelet. The wavelet-transformed scalograms were resized to 224 × 224 for normalization. Then classification was performed by using three models of deep learning.

**Figure 8:**
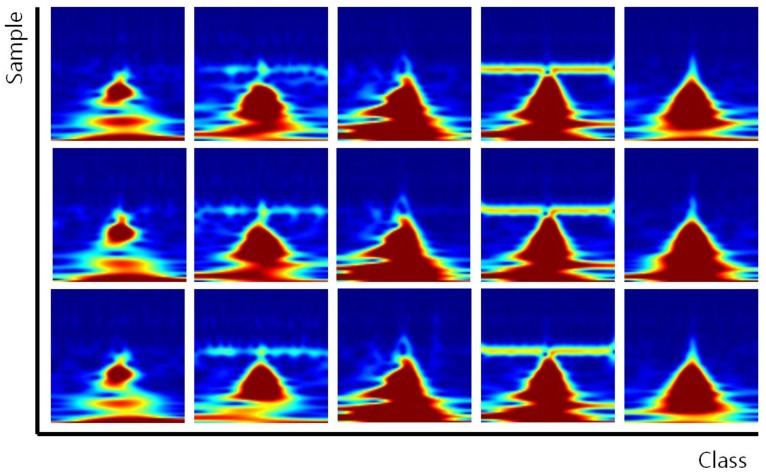
Scalograms of PTB-ECG.

In the case of PTB-ECG, the 79 people consisting of small R-peak points were abandoned among 290 people. The number of people is the number of classes to be identified. The common maximum count of R-peak points among 211 people was 120. Therefore, the database consisted of 120 data per class. The size of the constructed database was 784 × 25,320 (120 data/class × 211 classes); the row presents the dimension of the data, while the column presents the number of data. The sizes of the data for training and test were 784 × 12,660 each, because the ratio of training was 50%, and then the 1D signals were transformed to scalograms. Figure 8 shows the scalograms of PTB-ECG.

Simple CNN was tested as conventional method. It is composed of an input layer, three convolutional layers, two max-pooling layers, and one fully-connected layer. In first layer, an input layer takes 28 × 28 × 3 image. In the second layer, a convolutional layer with 3, 8, and 1 as the filter size, number of filters, and number of padding size extracts features, respectively. In the third layer, a max-pooling layer with 2, 2 as filter size, stride is performed. In the fourth layer, a convolutional layer with 3, 16, and 1 as the filter size, number of filters, and number of padding size extracts features, respectively. In the fifth layer, a max-pooling layer with 2, 2 as filter size, stride is performed. In the sixth layer, a convolutional layer with 3, 32, and 1 as the filter size, number of filters, and number of padding size extracts features, respectively. Finally, the fully-connected layer and softmax layer are performed.

**Figure 9:**
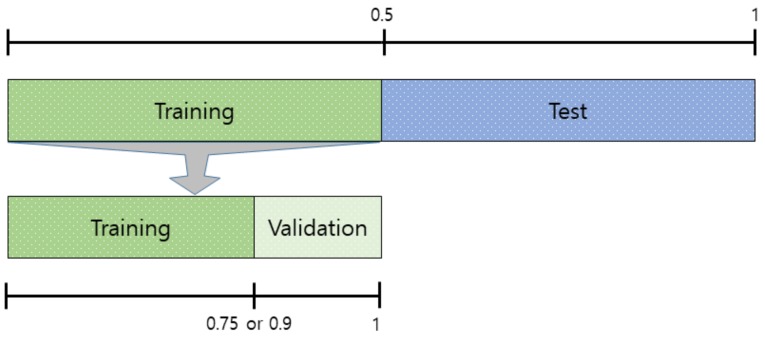
Data of training, validation, and test.

**Figure 10:**
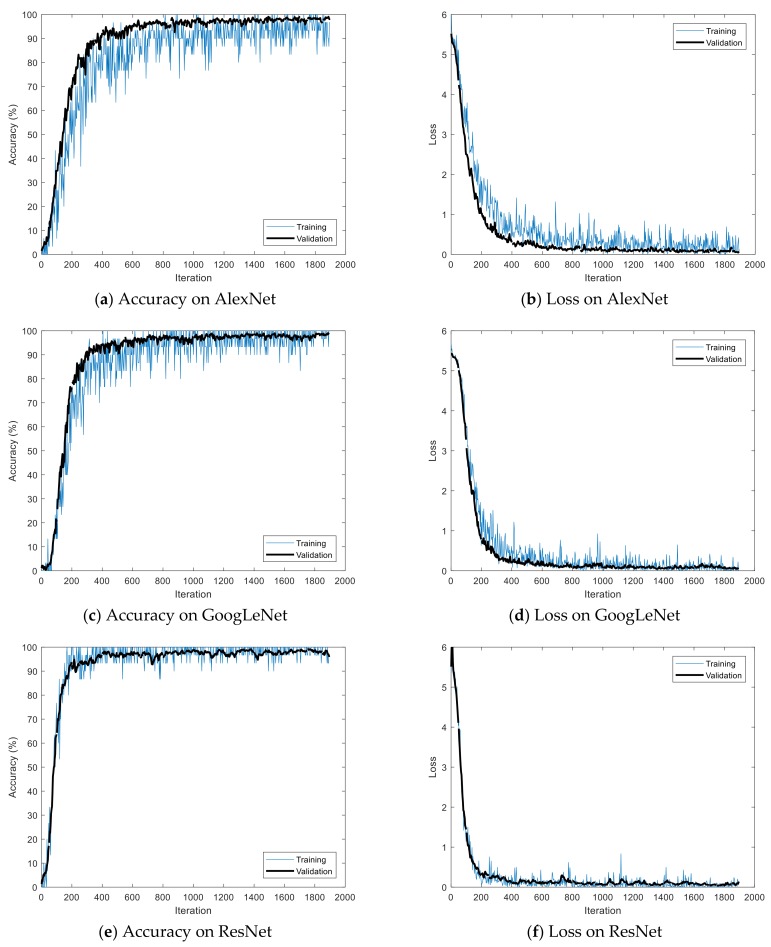
Training processes in PTB-ECG.

**Figure 11:**
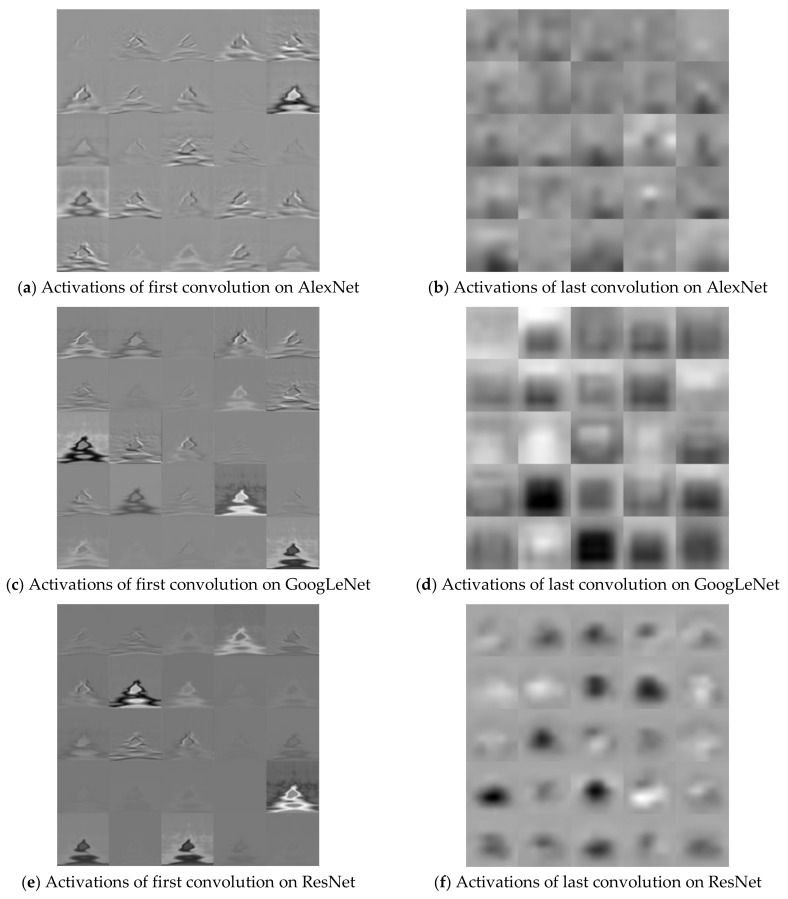
Comparison of the activations of the first and last convolutions in PTB-ECG.

**Figure 12:**
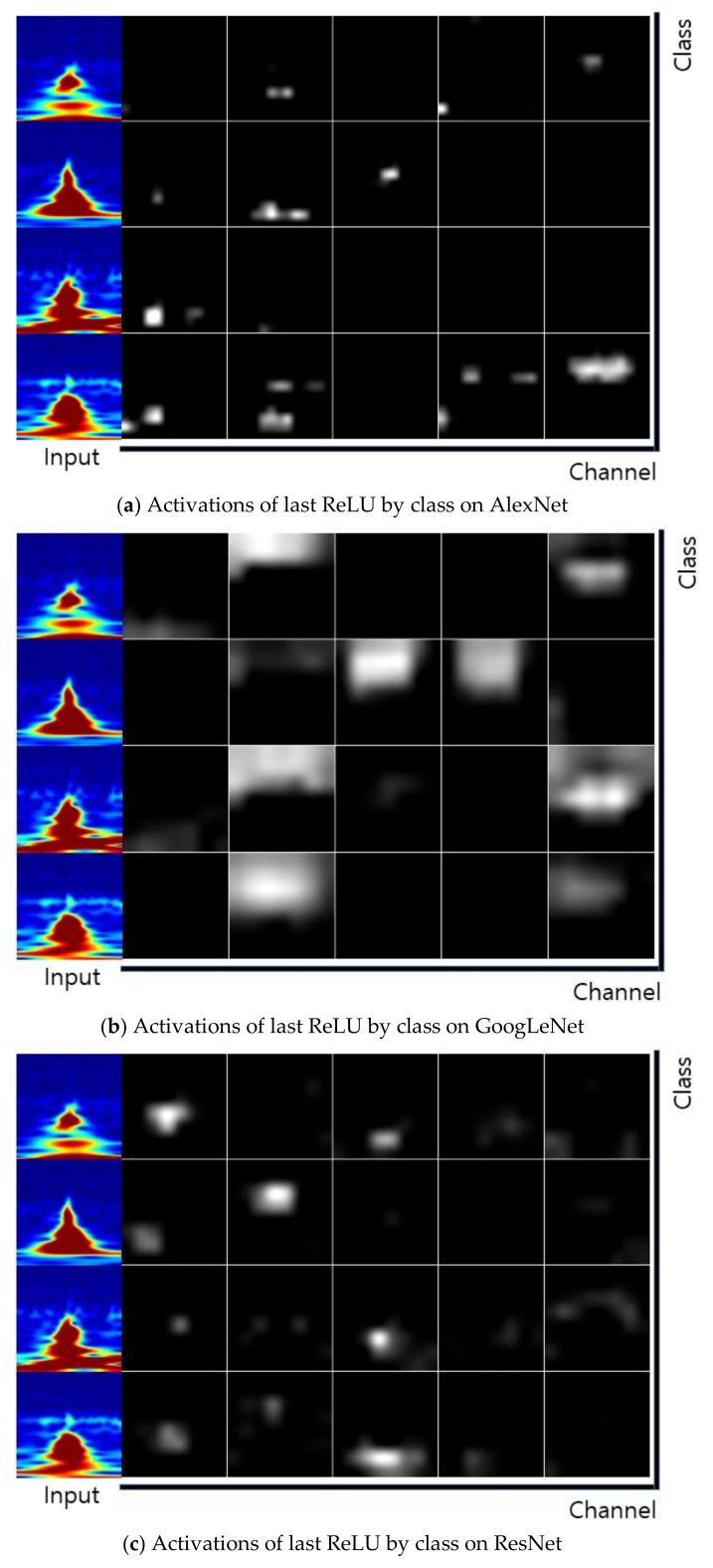
Comparison of the activations of the last ReLU (Rectified Linear Unit) by class in PTB-ECG.

The Stochastic Gradient Descent Method (SGDM), RMSProp, and Adaptive Moment Estimation (Adam) were used as learning methods. The basic learning method is a SGDM that moves in the opposite direction to the derivative direction from a group of random samples. Learning methods derived from SGDM are RMSProp and Adam. Their main strategy is to update parameters with a moderate learning rate at the beginning of learning and to reduce the learning rate as they approach the solution. Adam combines the advantages of momentum and RMSProp. In general, the loss function oscillates up and down, but it decreases as a whole during learning. The momentum prevents the current gradient from changing too much using the past gradient [53]. The initial learning rate was 0.0001, and the recognition rate was confirmed by changing the size of minibatch. The multiplier for the learning rate was the multiplication of the learning rate for the weight and bias of the last fully connected layer. The epochs of learning were applied 5, 10, and 20 times. The range of transferred units, whether to import parameter values of the already learned deep learning model or not, was specified by transferred units; the X means learning without transfer. The indices of the last unit of AlexNet, GoogLeNet, and ResNet were 25, 144, and 347, respectively. Training data was divided into training and validation data again. Figure 9 shows data of training, validation, and test. The validation accuracy is a recognition rate for confirming the learning state during training. It helps determine whether overfitting occurs or not. If the difference of accuracy between training and validation is large, it means overfitting occurs. The test accuracy is a recognition rate when new data is input after the completion of learning. Recently, Lee proposed EECGNet for personal identification. For comparing performance with the state of the art, an additional experiment is performed using EECGNet with input of scalogram. We used patch size (K), the number of filters (L), block size (h), and overlap ratio of block (R) as parameters of EECGNet. For more detail is in Lee [54]. Table 1, Table 2, Table 3, Table 4 and Table 5 show the accuracies of simple CNN, AlexNet, GoogLeNet, ResNet, and EECGNet on PTB-ECG, respectively. In the case of PTB-ECG, the highest accuracy of simple CNN was 86.02% when the training method, minibatch size, transferred units, and epoch were (Adam, 200, X, and 13), respectively; the highest accuracy of AlexNet was 97.37% when the training method, minibatch size, transferred units, and epoch were (Adam, 100, X, and 20), respectively; the highest accuracy of GoogLeNet was 97.83% when the training method, minibatch size, transferred units, and epoch were Adam, 30, X, and 20, respectively; the highest accuracy of ResNet was 98.10% when the training method, minibatch size, transferred units, and epoch were RMSProp, 30, X, and 10, respectively. The ResNet was 0.73%—0.27% higher than AlexNet or GoogLeNet on PTB-ECG. Figure 10 shows the training processes in PTB-ECG. Figure 11 shows a comparison of the activations of the first and last convolutions in PTB-ECG. Figure 12 shows a comparison of the activations of the last ReLU by class in PTB-ECG. Figure 10, Figure 11 and Figure 12 were recorded when the multiplier of learning rate, minibatch size, transferred units, and epoch were 10, 30, X, and 5, respectively, in training.

**Figure 13:**
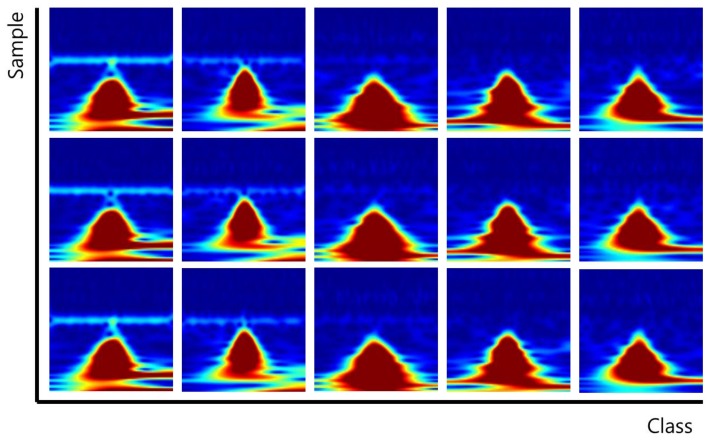
Scalograms of CU-ECG.

**Figure 14:**
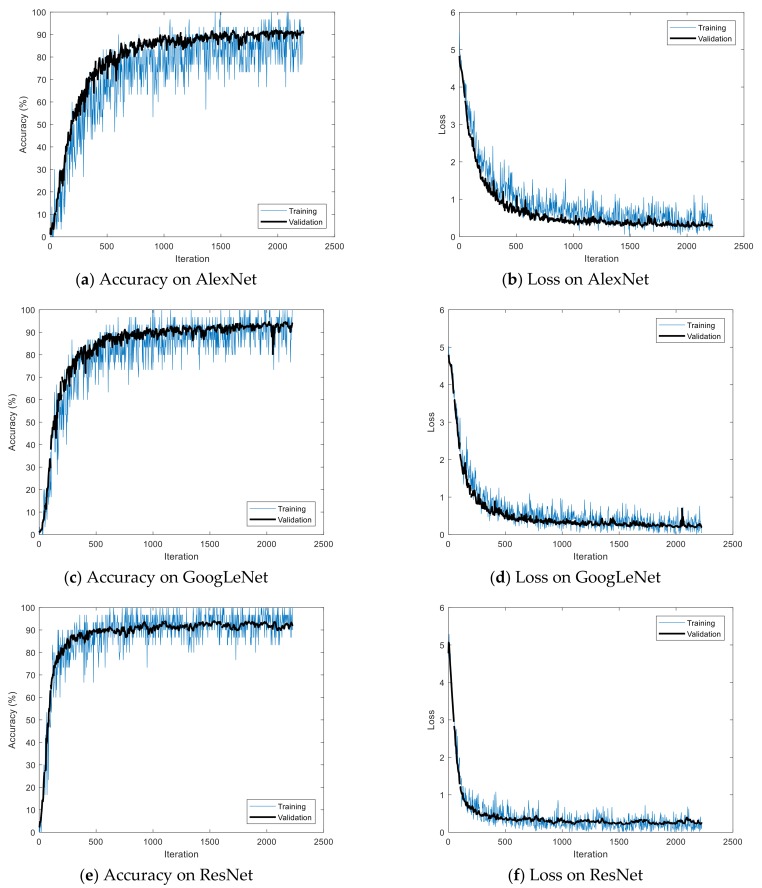
Training processes in CU-ECG.

**Figure 15:**
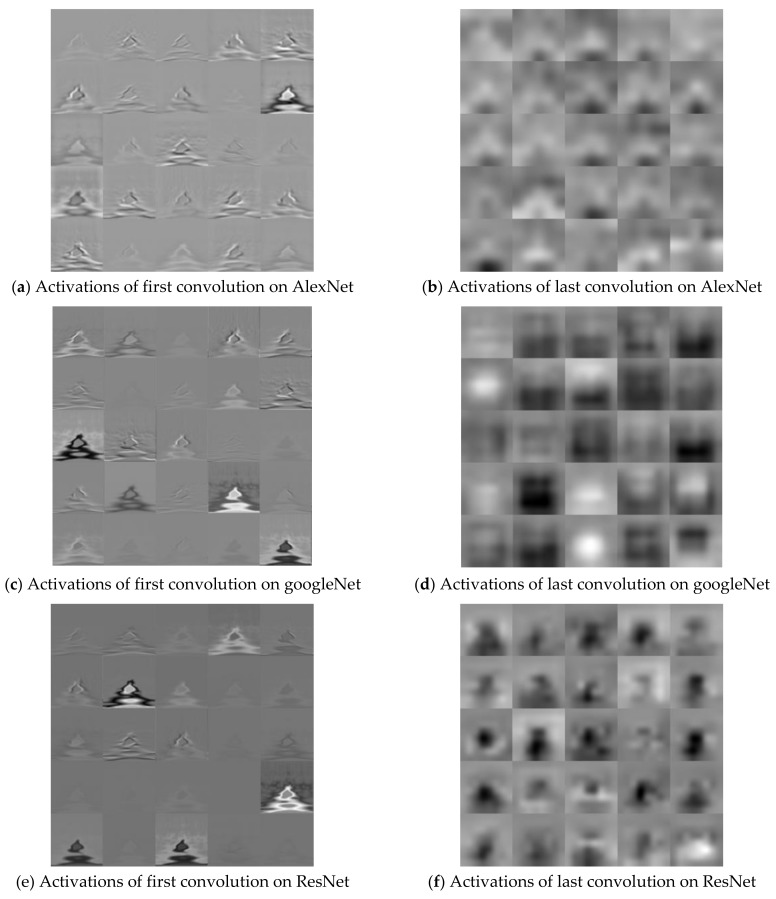
Comparison of the activations of the first and last convolutions in CU-ECG.

**Figure 16:**
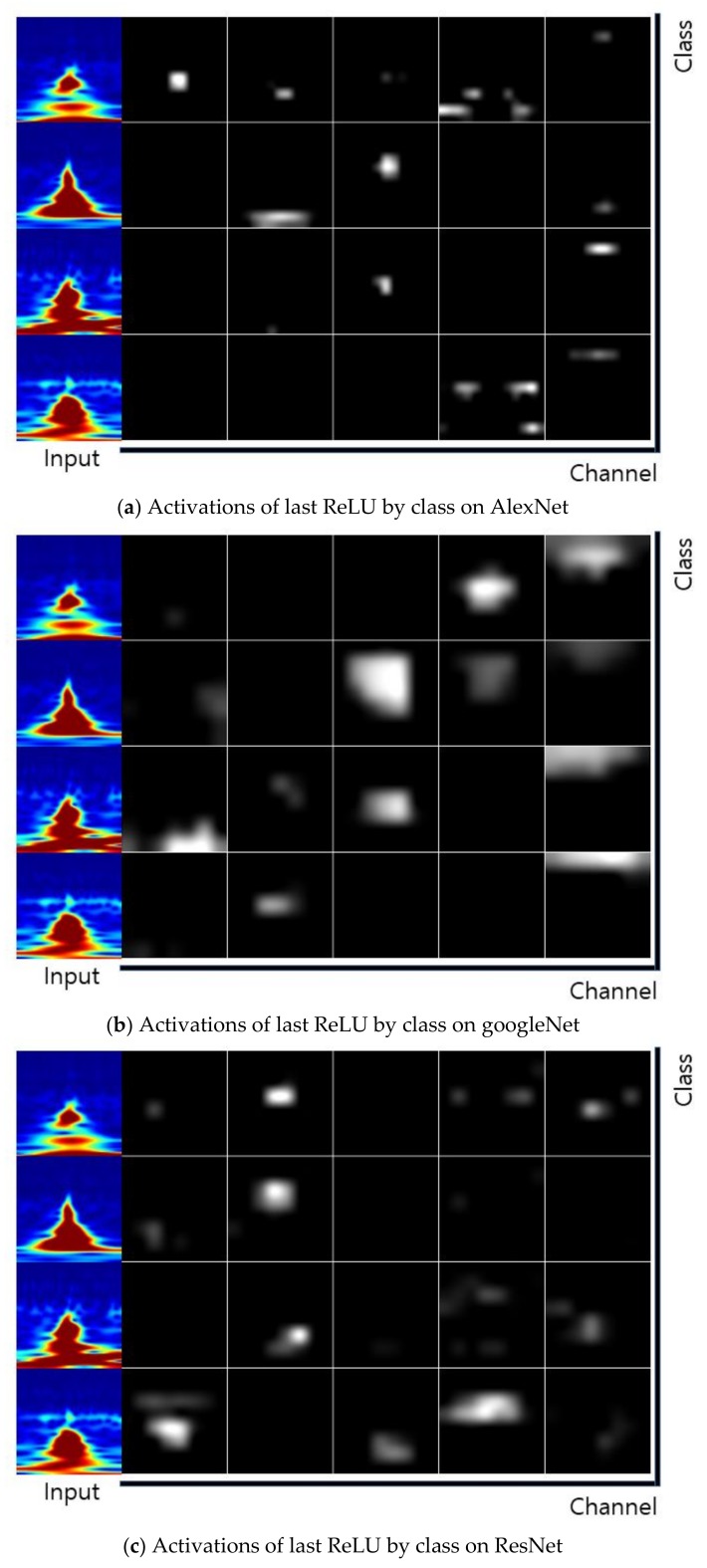
Comparison of the activations of the last ReLU by class in CU-ECG.

In the case of CU-ECG, the database was resampled from 500 to 1 kHz, because the data took much memory and processing load, and one person consisting of a small number of R-peak points was abandoned among the 100 people. The number of people is the number of classes to be identified. The common maximum count of R-peak points among 99 classes was 300. Therefore, the database consisted of 300 data per class. The size of the constructed database was 784 × 29,700 (300 data/class × 99 classes); the row presents the dimension of the data, while the column presents the number of data. The sizes of the data for training and test were 784 × 14,850 each, because the ratio of training was 50%, and then the 1D signals were transformed to scalograms. Figure 13 shows the scalograms of CU-ECG. The initial learning rate was 0.0001, and the recognition rate was confirmed by changing the size of minibatch. The epochs of learning were applied 5, 10, and 20 times. Table 6, Table 7, Table 8, Table 9 and Table 10 show the accuracies of simple CNN, AlexNet, GoogLeNet, ResNet, and EECGNet on CU-ECG, respectively. In the case of CU-ECG, the highest accuracy of simple CNN was 60.83% when the training method, minibatch size, transferred units, and epoch were Adam, 200, X, and 8, respectively; the highest accuracy of AlexNet was 92.26% when the training method, minibatch size, transferred units, and epoch were Adam, 30, X, and 20, respectively; the highest accuracy of googleNet was 93.08% when the training method, minibatch size, transferred units, and epoch were Adam, 30, X, and 20, respectively; the highest accuracy of ResNet was 93.20% when the training method, minibatch size, transferred units, and epoch were RMSProp, 30, X, and 10, respectively. The ResNet was 0.94%—0.12% higher than AlexNet or googleNet on CU-ECG. Figure 14 shows the training processes in CU-ECG. Figure 15 shows a comparison of the activations of the first and last convolutions in CU-ECG. Figure 16 shows a comparison of the activations of the last ReLU by class in CU-ECG. Figure 14, Figure 15 and Figure 16 were recorded when the multiplier of learning rate, minibatch size, transferred units, and epoch were 10, 30, X, and 5, respectively, in training.

**Figure 17:**
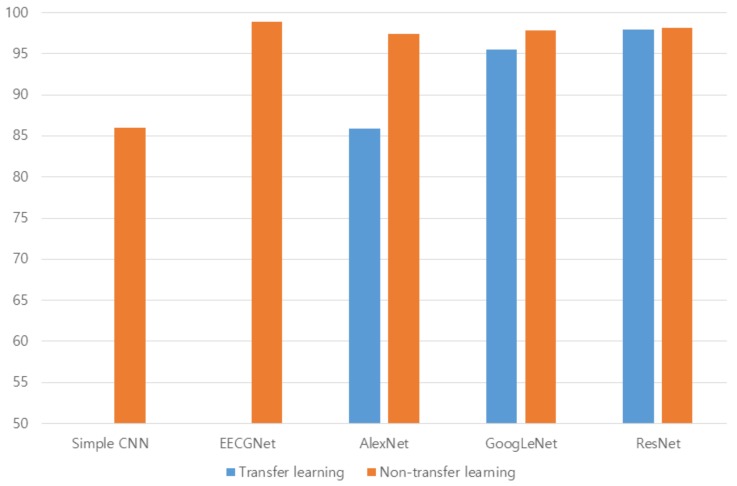
Comparison of the highest accuracies between transfer and nontransfer learning on PTB-ECG.

**Figure 18:**
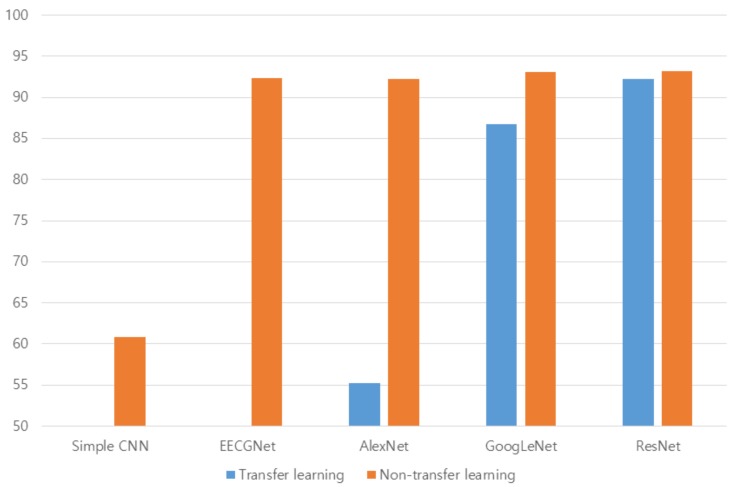
Comparison of the highest accuracies between transfer and nontransfer learning on CU-ECG.

Figure 17 shows a comparison of the highest accuracies between transfer and nontransfer learning on PTB-ECG. Figure 18 shows a comparison of the highest accuracies between transfer and nontransfer learning on CU-ECG. The accuracies were quite different among models trained with transfer, but the accuracies were slightly different among models trained without transfer. Models trained with transfer were optimized for previous training data. So, the large difference of accuracy between transfer and nontransfer means that the model is sensitive to training data. The differences of AlexNet, googleNet, and ResNet were 11.53, 2.32, and 0.15%, respectively, on PTB-ECG, and the differences of AlexNet, googleNet, and ResNet were 37.05, 6.37, and 0.99%, respectively, on CU-ECG. The ResNet has 11.38% and 2.17% lower differences between transfer and nontransfer, respectively, than AlexNet and googleNet on PTB-ECG. The ResNet has 36.06% and 5.38% lower differences between transfer and nontransfer, respectively, than AlexNet and googleNet on CU-ECG. Table 11 indicates comparison of computational time. The training times were measured during one epoch when the training method, minibatch size, and transferred units were Adam, 64, and X, and the test times were measurements on one signal processing.

**Figure 19:**
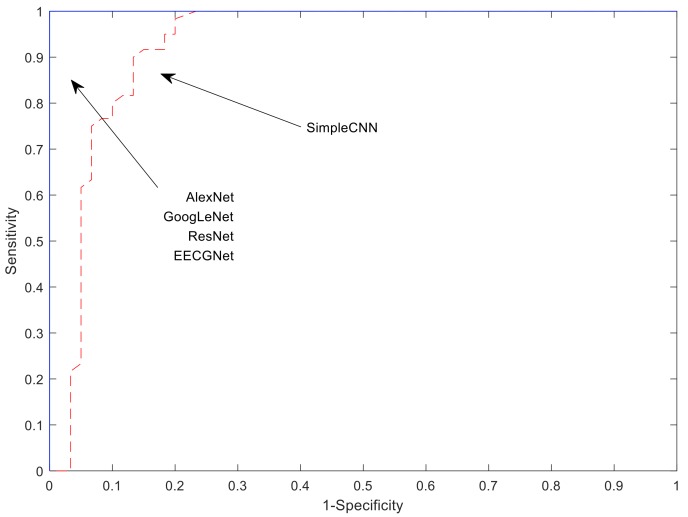
Comparison of the ROC (Receiver Operating Characteristic) curves on PTB-ECG.

To measure performance of verification, the data is reconstructed into two classes of one-against-all. To balance the two sample sets in the case of PTB-ECG, only 61 people among 211 people were used because a person has 60 data. The first person is positive and the people from 2 to 61 are negative. The negative samples are constructed by taking one sample per person. The models are trained with training method, minibatch size, and transferred units as Adam, 60, and X. Only ResNet was 10 as minibatch size. K, L, h, and R were 8, 8, 8, and 0.5 in EECGNet, respectively. The performances are measured on test dataset. Our research focused on individual identification. Table 12 shows verification performance on PTB-ECG. Figure 19 describes comparison of the ROC(Receiver Operating Characteristic) curves on PTB-ECG.

## 5. Conclusions

We performed comparative analysis of deep models in biometrics using scalogram of electrocardiogram. Since biometrics using ECG signals are sensitive to noise, studies have been conducted by transforming signals into the frequency domain that is efficient for analyzing noisy signals. By transforming the signal from the time domain to the frequency domain using the wavelet, the 1-D signal becomes a 2-D matrix, and it could be analyzed at multiresolution. However, this process makes for a morphologically complex signal analysis. This means that existing simple classifiers could be poor in performance. We investigate the possibility of using the scalogram of electrocardiogram as input to CNN of deep learning, which exhibits optimal performance for the classification of morphological imagery. AlexNet, GoogLeNet, and ResNet are considered deep models of the convolutional neural network in this paper. The experiments are performed on two databases for performance evaluation. The ResNet was 0.73%—0.27% higher than AlexNet or googleNet on PTB-ECG—and the ResNet was 0.94%—0.12% higher than AlexNet or GoogleNet on CU-ECG. The ResNet had 11.38% and 2.17% lower differences between transfer and nontransfer, respectively, than AlexNet and googleNet on PTB-ECG. The ResNet had 36.06% and 5.38% lower differences between transfer and nontransfer, respectively, than AlexNet and googleNet on CU-ECG. For further research, we will study less influenceable ECG representation to noise.

## Figures and Tables

**Table 1:** Performance of simple CNN (convolutional neural network) on PTB-ECG.

Training Method	Ratio of Training and Validation	Minibatch Size	Multiplier of Learning Rate	Epoch	Transferred Units	Validation Accuracy	Test Accuracy
SGDM	90:10	30	10	5	X	18.56	18.05
RMSProp	90:10	30	10	2	X	41.07	39.11
Adam	90:10	20	10	2	X	48.66	46.54
Adam	90:10	30	10	2	X	58.53	56.11
Adam	90:10	40	10	3	X	61.14	59.39
Adam	90:10	50	10	3	X	74.09	71.22
Adam	90:10	64	10	6	X	86.73	83.69
Adam	90:10	100	10	8	X	87.05	85.10
Adam	90:10	200	10	10	X	86.65	83.56
Adam	90:10	200	10	13	X	89.34	86.02

**Table 2:** Performance of AlexNet on PTB-ECG.

Training Method	Ratio of Training and Validation	Minibatch Size	Multiplier of Learning Rate	Epoch	Transferred Units	Validation Accuracy	Test Accuracy
SGDM	90:10	30	10	5	1–22	75.99	72.74
SGDM	90:10	30	10	20	1–22	86.73	84.57
RMSProp	90:10	30	10	5	1–22	64.30	63.17
RMSProp	90:10	30	10	20	1–22	58.29	55.70
Adam	75:25	20	10	5	1–22	63.38	61.97
Adam	75:25	30	10	5	1–22	68.94	67.14
Adam	50:50	30	10	10	X	97.54	95.77
Adam	75:25	30	10	20	1–22	85.34	84.12
Adam	90:10	30	10	20	X	98.89	97.37
Adam	75:25	40	10	20	1–22	85.47	83.64
Adam	90:10	50	10	20	1–22	86.10	83.73
Adam	90:10	100	10	20	1–22	87.28	85.84

**Table 3:** Performance of googleNet on PTB-ECG.

Training Method	Ratio of Training and Validation	Minibatch Size	Multiplier of Learning Rate	Epoch	Transferred Units	Validation Accuracy	Test Accuracy
SGDM	90:10	30	10	5	1–110	89.81	86.94
SGDM	90:10	30	10	20	1–110	95.02	94.40
RMSProp	90:10	30	10	5	1–110	97.16	94.45
RMSProp	90:10	30	10	20	1–110	97.39	94.94
Adam	90:10	30	10	5	X	97.87	95.14
Adam	90:10	30	10	5	1–110	96.21	93.96
Adam	90:10	30	10	10	1–110	96.37	94.48
Adam	50:50	30	10	10	X	97.35	95.50
Adam	90:10	30	10	20	1–110	98.10	95.51
Adam	90:10	30	10	20	X	99.37	97.83
Adam	90:10	40	10	5	1–110	95.10	93.84
Adam	90:10	50	10	5	1–110	94.63	92.03
Adam	90:10	100	10	5	1–110	95.58	93.38

**Table 4:** Performance of ResNet on PTB-ECG.

Training Method	Ratio of Training and Validation	Minibatch Size	Multiplier of Learning Rate	Epoch	Transferred Units	Validation Accuracy	Test Accuracy
SGDM	90:10	30	10	5	1–310	93.68	91.97
SGDM	90:10	30	10	10	1–310	98.03	96.28
RMSProp	90:10	30	10	5	1–310	98.97	97.19
RMSProp	90:10	30	10	10	1–310	99.61	97.95
RMSProp	90:10	30	10	10	X	99.37	98.10
Adam	90:10	30	10	5	1–310	98.42	96.11
Adam	90:10	30	10	10	1–310	98.66	96.96
Adam	50:50	30	10	5	X	96.82	95.07
Adam	50:50	30	10	10	X	97.30	95.69
Adam	90:10	30	10	10	X	99.13	97.60
Adam	90:10	40	10	5	1–310	98.34	96.48
Adam	90:10	50	10	5	1–310	98.10	96.14
Adam	90:10	100	10	5	1–310	98.34	96.79

**Table 5:** Performance of EECGNet on PTB-ECG.

K	L	h	R	Training Accuracy	Test Accuracy
4	4	4	0.5	99.78	98.89
8	4	8	0.5	99.72	98.04
12	8	12	0.5	99.72	98.15

**Table 6:** Performance of simple CNN on CU-ECG.

Training Method	Ratio of Training and Validation	Minibatch Size	Multiplier of Learning Rate	Epoch	Transferred Units	Validation Accuracy	Test Accuracy
SGDM	90:10	30	10	3	X	19.73	18.90
RMSProp	90:10	30	10	1	X	27.00	24.55
Adam	90:10	20	10	1	X	20.27	19.75
Adam	90:10	30	10	1	X	26.60	23.86
Adam	90:10	40	10	2	X	45.52	41.91
Adam	90:10	50	10	2	X	47.34	44.30
Adam	90:10	64	10	2	X	39.33	37.24
Adam	90:10	100	10	4	X	49.43	46.38
Adam	90:10	200	10	6	X	56.16	51.14
Adam	90:10	200	10	8	X	67.34	60.83

**Table 7:** Performance of AlexNet on CU-ECG.

Training Method	Ratio of Training and Validation	Minibatch Size	Multiplier of Learning Rate	Epoch	Transferred Units	Validation Accuracy	Test Accuracy
SGDM	90:10	30	10	5	1–22	47.21	44.05
SGDM	90:10	30	10	20	1–22	52.66	51.36
RMSProp	90:10	30	10	5	1–22	33.47	30.82
RMSProp	90:10	30	10	20	1–22	31.78	28.26
Adam	90:10	30	10	5	1–22	47.57	43.95
Adam	50:50	30	10	10	X	86.84	81.87
Adam	90:10	30	10	10	1–22	54.01	49.58
Adam	90:10	30	10	20	1–22	56.30	53.04
Adam	90:10	30	10	20	X	94.28	92.26
Adam	90:10	40	10	20	1–22	58.05	55.21
Adam	90:10	50	10	20	1–22	56.23	53.58
Adam	90:10	100	10	20	1–22	56.50	53.64

**Table 8:** Performance of googleNet on CU-ECG.

Training Method	Ratio of Training and Validation	Minibatch Size	Multiplier of Learning Rate	Epoch	Transferred Units	Validation Accuracy	Test Accuracy
SGDM	90:10	30	10	5	1–110	64.11	62.16
SGDM	90:10	30	10	20	1–110	81.75	78.45
RMSProp	90:10	30	10	5	1–110	85.05	70.79
RMSProp	90:10	30	10	20	1–110	89.70	84.94
Adam	90:10	30	10	5	1–110	82.76	79.55
Adam	90:10	30	10	10	1–110	89.23	85.12
Adam	50:50	30	10	10	X	88.46	83.75
Adam	90:10	30	10	20	1–110	89.83	86.71
Adam	90:10	30	10	20	X	95.35	93.08
Adam	90:10	30	20	10	1–110	89.23	84.68
Adam	90:10	40	10	5	1–110	84.65	81.23
Adam	90:10	50	10	5	1–110	82.69	79.58
Adam	90:10	100	10	5	1–110	81.41	76.57

**Table 9:** Performance of ResNet on CU-ECG.

Training Method	Ratio of Training and Validation	Minibatch Size	Multiplier of Learning Rate	Epoch	Transferred Units	Validation Accuracy	Test Accuracy
SGDM	90:10	30	10	5	1–310	85.86	82.25
SGDM	90:10	30	10	10	1–310	91.38	88.19
RMSProp	90:10	30	10	5	1–310	93.20	90.79
RMSProp	90:10	30	10	10	1–310	94.34	91.93
RMSProp	90:10	30	10	10	X	94.75	93.20
Adam	90:10	30	10	5	1–310	93.87	90.88
Adam	50:50	30	10	5	X	91.00	86.58
Adam	90:10	30	10	10	1–310	94.95	92.21
Adam	90:10	30	10	20	1–310	94.28	91.47
Adam	90:10	30	20	10	1–310	93.06	90.67
Adam	90:10	30	30	10	1–310	93.33	89.93
Adam	90:10	30	10	10	X	94.21	91.74
Adam	90:10	40	10	5	1–310	91.25	89.45
Adam	90:10	50	10	5	1–310	89.90	87.74
Adam	90:10	100	10	5	1–310	93.20	90.88

**Table 10:** Performance of EECGNet on CU-ECG.

K	L	h	R	Training Accuracy	Test Accuracy
4	4	4	0.5	95.92	92.29
8	4	8	0.5	95.72	86.96
12	8	12	0.5	95.88	89.50

**Table 11:** Comparison of computational time.

Model	Training Time		Test Time
	PTB-ECG	CU-ECG	
Simple CNN	1 m 15 s	1 m 33 s	0.000423 s
AlexNet	3 m 38 s	4 m 26 s	0.001809 s
GoogLeNet	9 m 11 s	11 m 37 s	0.004704 s
ResNet	77 m 28 s	92 m 36 s	0.012437 s
EECGNet	31 m 53 s	36 m 1 s	0.295800 s

**Table 12:** Verification performance on PTB-ECG.

Model	Accuracy (%)	Sensitivity	Specificity	FPR	FNR	EER (%)
Simple CNN	87.50	0.95	0.80	0.20	0.05	13.33
AlexNet	92.50	0.85	1.00	0.00	0.15	0.00
GoogLeNet	100.00	1.00	1.00	0.00	0.00	0.00
ResNet	99.17	1.00	0.9833	0.02	0.00	0.00
EECGNet	100.00	1.00	1.00	0.00	0.00	0.00
